# Estimating the energy consumption for residential buildings in semiarid and arid desert climate using artificial intelligence

**DOI:** 10.1038/s41598-024-63843-w

**Published:** 2024-06-13

**Authors:** Hossam Wefki, Rana Khallaf, Ahmed M. Ebid

**Affiliations:** 1 Civil Engineering Department, Port Said University, Port Fouad, 42526 Egypt; 2 Structural Engineering and Construction Management Department, Future University in Egypt, Cairo, 11835 Egypt

**Keywords:** Building energy consumption, Artificial intelligence, Energy efficiency, Engineering, Civil engineering

## Abstract

This research aims to develop predictive models to estimate building energy accurately. Three commonly used artificial intelligence techniques were chosen to develop a new building energy estimation model. The chosen techniques are Genetic Programming (GP), Artificial Neural Network (ANN), and Evolutionary Polynomial Regression (EPR). Sixteen energy efficiency measures were collected and used in designing and evaluating the proposed models, which include building dimensions, orientation, envelope construction materials properties, window-to-wall ratio, heating and cooling set points, and glass properties. The performance of the developed models was evaluated in terms of the RMS, R^2^, and MAPE. The results showed that the EPR model is the most accurate and practical model with an error percent of 2%. Additionally, the energy consumption was found to be mainly governed by three factors which dominate 87% of the impact; which are building size, Solar Heating Glass Coefficient (SHGC), and the target inside temperature in summer.

## Introduction

Energy is an essential aspect of sustainability due to its increasing consumption worldwide and its contribution to climate change and environmental concerns. One of the critical measures toward efficient energy production, distribution, and consumption is accurately forecasting the energy consumption in buildings. These buildings represent a large portion of the energy consumption and potential for reduction^1^. During the past few decades, the demand for improving building energy performance has increased due to population growth as well as global economic growth worldwide. The global building sector consumes an estimated 36% of the global energy in the form of electricity, gaseous, liquid, and solid fuels, and district energy for building uses (e.g., heating, cooling, lighting, and equipment)^2^, and is responsible for around 27% of global operational related CO_2_ emissions. The construction and building sectors accountsfor nearly 38% of carbon emissions globally^3^. Buildings are responsible for a large percentage of the CO_2_ emissions worldwide, ranging from 27% in Australia to 40% in the United States and 46% in the United Kingdom^4^. According to^5^, about a quarter of the world’s energy is consumed in residential buildings. However, according to^6^, the commercial, industrial, and transportation sectors have been widely studied due to public and economic interests. The building sector, on the other hand, has not provided similarfinancial incentives that would drive its study.

Energy demand can be affected by several factors. A heatwave can make the building envelope hotter and induce additional load on the Heating, Ventilation, and Air Conditioning (HVAC) system. Moreover, the energy demand associated with space cooling in buildings has almost tripled since the 1990s^7^. Several methods exist to mitigate problems stemming from energy consumption and to decrease the amount of greenhouse gases. These include methods to calculate energy consumption^8^, optimize energy consumption^9^, or methods to evaluate design alternatives^1^. Hence, improving the energy properties of buildings can be an effective strategy for lowering the energy demand and enhancing the indoor air quality. In order to do so, correct prediction of energy usage and evaluation of alternatives is needed^10^.

Energy efficiency of residential buildings can be improved in several ways such as selecting construction materials with low thermal conductivity (U-value)^11,12^. Another way would be to focus on estimating the energy consumption during the design stages by generating several alternative design options. This is especially beneficial during the early design stages where decisions that are made can significantly impact a building’s performance. For instance, selecting the correct building orientation and suitable shape can cause a reduction in the energy consumption by 30–40% even without any additional cost^13^. Building design procedures considering energy efficiency are complex multi-criteria problems where the interdependency among design parameters can be difficult to assess without the use of simulation engines. The uncertainty and complexity of building performance design makes it difficult to make decisions solely based on experience, therefore, models are essential. Architects conduct studies and estimations to evaluate the influence of individual parameters on the energy efficiency. They expend 40%—50% of their time developing designs and using available tools for energy simulations to justify the specified design, only to explore a few limited alternatives. Several processes and tools available in current practice have limitations that result in narrow design space explorations^14^. An integrated approach is recommended to evaluate multiple design alternatives to increase building energy performance. This approach needs to consider all contributing factors that affect building energy consumption such as floorplan, size, and number of windows^15^.

Simulation-based artificial intelligence techniques commonly require hundreds or thousands of simulation runs. In order to obtain dependable outcomes, it is important to compute the energy consumption of every design choice by constructing a comprehensive building model and employing simulation tools while taking into account the unique attributes of the building. Several parametric analyses are carried out by designers and architects to assess the significance of various energy efficiency factors. This research offers methods for estimating and predicting residential building energy consumption during the early design stages. In light of the fact that real measurements and simulation models requires time, which is costly; this study explores the application of artificial intelligence models for precise, effortless, and practical energy consumption estimation. Artificial intelligence methods and techniques can improve a model’s accuracy and computational capabilities. A vital component of these methods is the forecasting models implanted within and employed to estimate energy consumption. Multiple design alternatives are simulated using tools such as Rhinoceros/Grasshopper to generate energy consumption datasets that can then be used to build prediction models^13^. Therefore, this study investigates the use of artificial intelligence to develop different models to estimate the energy consumption values. For this purpose, three AI techniques are considered, namely Genetic Programming (GP), Artificial Neural Network (ANN), and Evolutionary Polynomial Regression (EPR) to improve the accuracy of the predictive models in developing a new building energy estimation model. The three models were developed, compared, and evaluated in estimating energy consumption of residential buildings during the early design stage. This study employed GP, ANN, and EPR for energy consumption estimation due to the distinct advantages these advanced AI techniques offer over traditional methods and other techniques. Unlike conventional approaches such as linear regression, which may struggle to capture the complex, non-linear relationships inherent in energy consumption data, GP evolves mathematical expressions to fit the data, enabling the discovery of nuanced interactions and patterns. Similarly, ANN, inspired by the human brain's structure, excels at learning from large and intricate datasets, allowing for the modeling of complex relationships among various factors influencing energy consumption. On the other hand, EPR combines the flexibility of polynomial regression with evolutionary optimization, enabling the discovery of optimal polynomial expressions while minimizing overfitting. These techniques offer a significant departure from traditional methods by providing the ability to capture non-linear relationships, adapt to diverse datasets, and produce accurate predictions. By leveraging the strengths of GP, ANN, and EPR, this study aims to develop more robust and flexible energy estimation models that can better inform decision-making in energy management, building design, and policy formulation, thus contributing to advancements in energy efficiency and sustainability in the built environment. The following section discusses the literature review on the proposed topic.

## Literature review

Previous literature has widely studied energy consumption and prediction due to its importance. There is a complex and somewhat intertwined relationship between the multiple variables that impact energy consumption associated with buildings. These variables also have nonlinear multivariate interrelationships. Therefore, it is essential to have procedures that can efficiently model the nonlinearities without expending a significant amount of time in the estimating process. Over the years, researchers have replaced traditional energy prediction methods with newer computational methods based on artificial intelligence techniques. These techniques are based on the data collected. The data-driven method is a sound technique to handle the complexity of building energy-related applications. However, the data collected on energy consumption is often very few or non-normal. The following subsections discuss the use of AI-based models for energy prediction from previous literature followed by a discussion of the different AI techniques.

### Artificial intelligence techniques for energy prediction

Several techniques exist for energy prediction modelling and simulation. This paper focuses on three of the commonly used methods, which are Genetic Programming (GP), Artificial Neural Network (ANN), and Evolutionary Polynomial Regression (EPR). Genetic Programming is a computational method that is developed as an extended optimization method based on Genetic Algorithms (GAs)^16^. It seeks to find an optimum global solution for a set of predictor input variables^17^. This is done by the automatic learning of programs and transforming and enhancing them into new ones^16^. Artificial Neural Networks (ANN) are one of the most commonly used techniques for building energy estimation and are known to model complex systems and solve nonlinear problems^3,18^. They are reported to be among the best techniques to efficiently model the input–output function associations and make estimates for unrecognized data^7^. They also have the ability to reduce run time and produce high accuracy in comparison to other methods^19^. ANNs have been reported as effective methods for building energy prediction due to their ability to perform nonlinear analysis^15^. According to literature review by^18^, they were found to have a higher accuracy over other methods. The third technique is Evolutionary polynomial regression (EPR), which is a data mining technique based on evolutionary computing that was developed to combine the power of numerical regression and genetic algorithms also to develop symbolic models^20^. It has mainly been used in applications for hydro informatics and other environment-related issues^21^. This paper presents an approach for improving the accuracy of energy consumption estimate during early design stage for residential buildings to encourage the design of efficient energy usage in buildings. The following section describes the methodology followed in this paper.

### Artificial intelligence models for energy prediction in literature

Several modelling approaches have been proposed to estimate energy consumption, including artificial intelligence techniques. Khan et al.^22^ proposed a framework to accurately predict the power consumption utilizing a hybrid AI model using historical data from building consumption. The trained model was evaluated on test data in terms of Mean Squared Error (MSE), Root Mean Squared Error (RMSE), and Mean Absolute Error (MAE). Runge and Saloux^23^ explored deep learning models to evaluate the future heating load in a district heating system, considering a prediction and a forecasting-based approach. The prediction model, which applied forecasted weather as an input, obtained a lower forecasting error than the forecasting-based approach. These approaches offered a better performance overall in estimating the future heating demand with an RMSE not exceeding 16% for all models.

Olu-Ajayi et al.^24^ presented a comparison between nine machine learning techniques for predicting annual energy consumption using a large dataset of residential buildings, which are: artificial neural network (ANN), Gradient Boosting (GP), Deep Neural Network (DNN), Random Forest (RF), Sticking, K Nearest Neighbour (KNN), Support Vector Machine (SVM), Decision Tree (DT), and Linear Regression (LR). The results showed that the DNN model produced better results for energy use prediction. Yang et al.^25^ proposed a combined deep learning approach using the Back Propagation Neural Network (BPNN), Extreme Gradient Boosting (XGBoost), and Long Short-Term Memory (LSTM) load forecasting model to accomplish heightened accuracy and load forecasting assuming a multi-time scale electricity consumption behaviour of single household subject users. Yazici et al.^19^ proposed a novel method based on Convolutional Neural Networks (CNNs) and Video Pixel Networks (VPNs) for short-time load forecasting, and conducetd a comparative analysis of the most prevalent deep learning architectures using a real-world case. The proposed model induced the best result in total, with a 2.21% mean absolute percentage error for predictions.

Jamei et al.^20^ developed a model to predict heating energy usage using data from a preliminary survey. The developed data was trained using support vector regression (SVR), multiple linear regression (MLR), and artificial neural network (ANN). ANN was reported to be slightly more accurate and faster than SVR and MLR. Li and Yao^26^ designed a framework to forecast the heating and cooling energy use of residential and non-residential buildings by developing an energy database to train different machine learning models. The results showed that the machine learning model can achieve accurate building Energy Use Intensity (EUI). Zou et al.^27^ employed the simulation-based method to predict the life cycle energy performance of residential buildings. The data is generated using a parametric simulation tool with Python to get future weather data files. The impact of passive design on energy performance and demand was also considered in varying climate and locations.

D'Amico et al.^28^ developed a decision support tool based on an artificial neural network that quickly and reliably determines the building energy performance. The ANN model established the relationships between input and output variables by generating reliable database representatives of Italian building stock with 28,080 records with satisfactory results and a high degree of reliability, emphasizing the use of ANN in solving the complexity of assessing building energy and environmental performance.

Mohammadi et al.^29^ proposed a prediction model based on a hybrid prediction engine and a new feature selection algorithm to enhance the empirical mode decomposition named sliding window bundled with an intelligent algorithm. The proposed model was combined with a novel shark smell optimization to increase the prediction accuracy by optimizing all weights to encounter adequate prediction consequences. Ullah et al.^30^ proposed a Hidden Markov model-based algorithm to predict energy consumption using dataset collected through smart meters. The proposed model was compared to ANN, SVM, and Classification and Regression Trees (CART) that produced a better result in terms of root mean square error. Fayaz and Kim^31^ proposed a methodology for energy consumption prediction in residential buildings utilizing real data using various deep learning methods. The results indicate that the performance of deep extreme learning machine (DELM) is far better than ANN and adaptive neuro-fuzzy interface system (ANFIS) for one week and one-month hourly energy prediction. Ascione et al.^32^ employed artificial neural networks to predict building energy performance with low computational time to assess energy consumption and occupants’ thermal comfort. Two families of ANNs were generated in MATLAB using the EnergyPlus simulation outcome as a target for networks’ training and testing. The regression coefficient and average relative error were acceptable values comparing the ANN’s prediction model with simulation engine targets.

Mocanu et al.^33^ investigated two newly invented stochastic models for time series of energy consumption forecast using benchmark datasets gathered from individual residential buildings. The ANNs, SVMs, RNNs, CRBMs, and FCRBMs models were used, evaluated, and compared to achieve the goal of energy prediction with high accuracy. Samuelson et al.^34^ presented a framework for the development of early-design guidance to inform architects and decision makers using parametric whole building energy simulation, performed 90,000+ energy simulations to generate an energy consumption dataset. Energy performance was found to be most sensitive to building orientation, window-to-wall ratio, and glass type. Fumo and Biswas^35^ performed single and multiple linear regression and quadratic regression analyses on hourly and daily data from a research house considering the outdoor temperature and solar radiation parameters. Results showed that multiple linear regression models presented an improved coefficient of determination, but deteriorated the root mean square error, accentuating the significance of utilizing both parameters to evaluate and compare models.

Fan et al.^36^ proposed a data mining-based approach to producing ensemble models for forecasting the next-day peak power demand and energy consumption. The ensemble models combine eight popular prediction algorithms, enabling the base models to complement each other and produce better generalization performance. The accuracies of the ensemble models were better than those of the base models. The support vector regression (SVR) and random forests (RF) had the most significant weights and gave the best performance. Elbeltagi and Wefki^37^ presented a model to predict the energy consumption for residential buildings during the early design stage using the power of ANNs. The data used for learning and testing the network was evaluated by calculating consumed energy from the simulation of multiple design options with random input parameters. Elhabyb et al.^38^ used multiple machine learning algorithms, mainly gradient boosting regressor (GBR), long short-term memory (LSTM), and random forest to predict energy consumption in educational buildings and compared between them.

## Methodology

**Figure 1:**
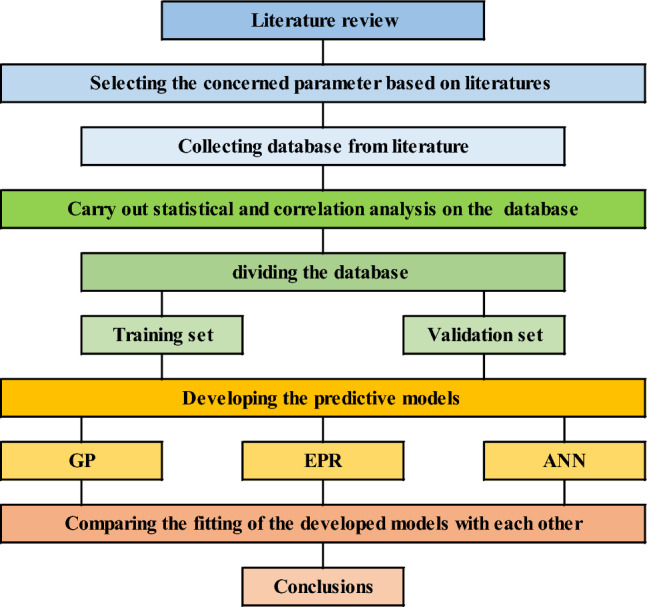
The used methodology flowchart.

In order to estimate the energy consumption, the methodology of this research starts with collecting, sorting, and analyzing data from previous research. The next step is to develop three predictive models to estimate the energy consumption (EC) values using building dimensions and orientation, envelope construction material properties, window sizes, glass properties, and finally, the desired inside temperature in both summer and winter. Three (AI) techniques are utilized, namely “Genetic programming” (GP), “Artificial Neural Network” (ANN), and “Evolutionary Polynomial Regression” (EPR). The performance of the developed models is evaluated in terms of the “Root of Mean Squared Error” (RMSE), “Coefficient of Determination” (R^2^), “Mean Error Percent” (Error%) and “Prediction accuracy percent” (Accuracy %) as shown in Eqs. (1) to (4)^4^. The following section describes the steps conducted in detail. Figure 1 summarizes the equations used in this study.

1 $$RMSE = \sqrt {\frac{1}{N}\mathop \sum \limits_{i = 1}^{N} \left( {y_{i} - \hat{y}} \right)^{2} }$$

2 $$Error \% = \frac{RMSE}{{\hat{y}}}$$

3 $$Accurcy \% = 1 - Error \%$$

4 $$R^{2} = 1 - \frac{{\sum \left( {y_{i} - \hat{y}} \right)^{2} }}{{\sum \left( {y_{i} - \overline{y}} \right)^{2} }}$$

### Collected database

A large database was obtained from a previous study^26^ and used in this research. This database contains the calculated energy consumptions (EC) for a number of buildings with different sizes, orientations, materials, and façades. The (EC) were calculated using Energy Plus package. Each record contains 17 data points as delineated below:

- Building dimensions: length (L), width (W) and height (H) in (m);
- Building orientation with respect to north (O) in (degrees);
- Windows to wall ratio for each façade, North (Wn), South (Ws), East (We) and West (Ww);
- Thermal conductivity (U-value) of walls (Uw), roofs (Ur), slab on grade (Us) in (W/m2K);
- Window glass properties, (U-value) (Ug) in (W/m2K), Solar Heat Gain Coefficient (SHGC) and Visible Transmittance percent (VT);
- The target temperature inside the building in winter (Heat) and in summer (Cool) in (oC);
- The calculated (EC) value in (Watts).

**Figure 2:**
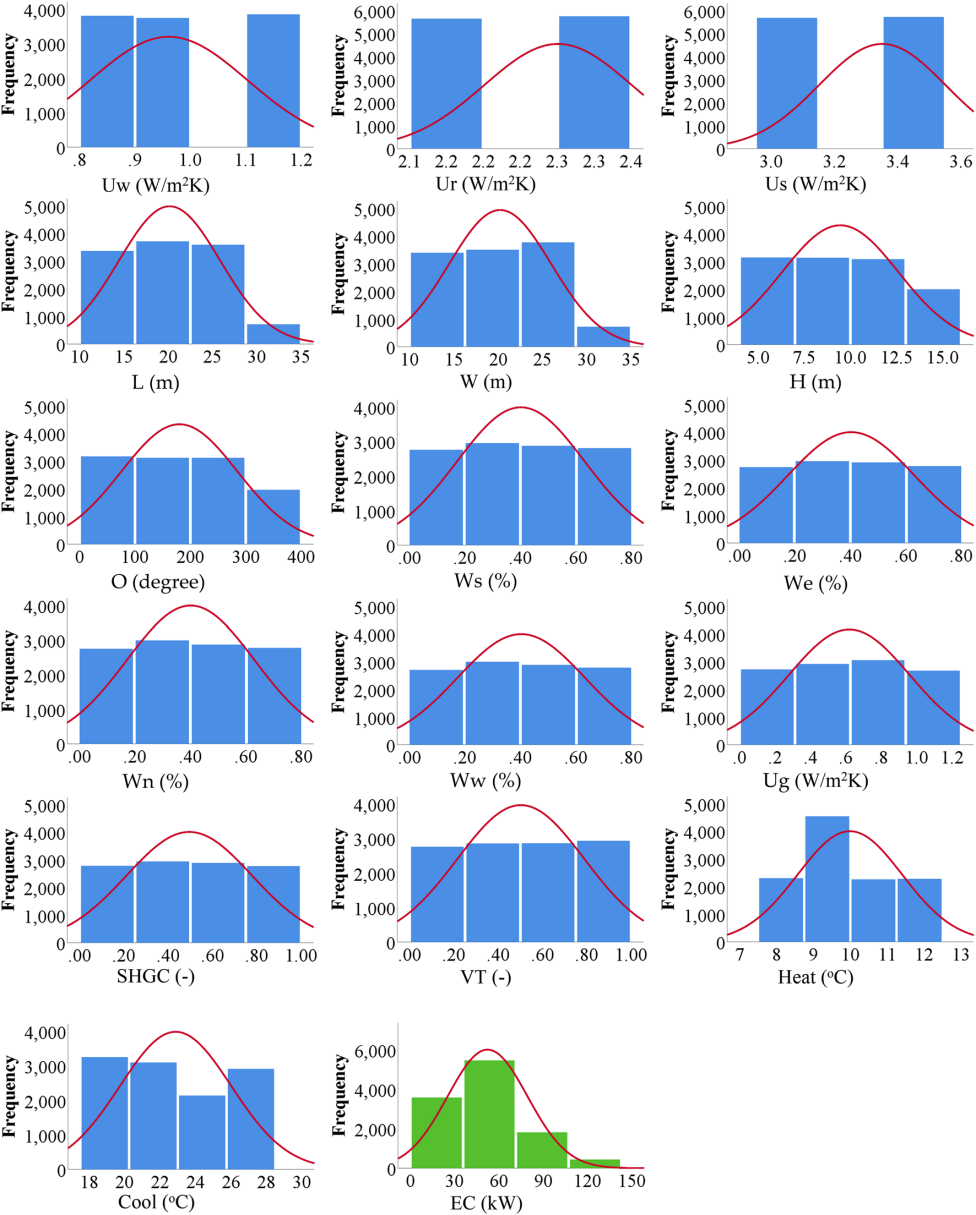
Frequency Distribution for input parameters (in blue) and output (EC) (in green).

A total of 11,500 records were available in the database. The database was randomly divided into 9000 records (≈78%) for training and 2500 records (≈22%) for validation. Table 1 summarizes both subsets statistically where the main target of this analysis is to make sure that for each considered parameter, both training and validations subsets have almost the same statistical characteristics. Hence, the validation subset could be used to monitor the over-fitting of the predictive models. Table 2 presents the inter-correlations between the considered parameters. The main goal of this table is to figure out how each parameter depends on the others and to make sure that all the considered parameters are independent so that there is no redundancy. The numbers showed that all considered parameters are independent (correlation coefficient <  < 1.0) and indicated a significant forward correlation between (EC) and (L, W, H, SHGC) and a reverse correlation with (Cool). Finally, the frequency distribution of each parameter is shown in Fig. 2 that shows that all considered parameters are spread along the considered range of each parameter so the developed predictive models will be valid all over the considered parameters’ ranges.

**Table 1:** Statistical characteristics of training and testing subsets.

	Min	Max	Range	Mean	S.D	Var	Skewness	Kurtosis
Training subset								
Uw	0.81	1.15	0.34	0.96	0.14	0.02	0.40	− 1.51
Ur	2.20	2.40	0.20	2.30	0.10	0.01	0.01	− 2.00
Us	3.15	3.55	0.40	3.35	0.20	0.04	− 0.02	− 2.00
L	10.00	30.00	20.00	19.96	5.75	33.01	0.02	− 1.18
W	10.00	30.00	20.00	20.01	5.80	33.65	− 0.01	− 1.22
H	4.00	15.00	11.00	9.60	3.15	9.92	− 0.04	− 1.18
O	0.00	360	360	180	105	11,014	− 0.01	− 1.22
Ws	0.00	0.80	0.80	0.40	0.23	0.05	− 0.01	− 1.19
We	0.00	0.80	0.80	0.40	0.23	0.05	− 0.02	− 1.20
Wn	0.00	0.80	0.80	0.40	0.23	0.05	0.01	− 1.20
Ww	0.00	0.80	0.80	0.40	0.23	0.05	0.00	− 1.19
Ug	0.01	1.20	1.19	0.62	0.34	0.12	− 0.08	− 1.19
SHGC	0.01	0.99	0.98	0.51	0.28	0.08	− 0.06	− 1.16
VT	0.01	0.99	0.98	0.50	0.29	0.08	0.00	− 1.21
Heat	8.00	12.00	4.00	9.99	1.42	2.01	0.01	− 1.31
Cool	18.00	28.00	10.00	22.94	3.16	9.96	0.01	− 1.22
EC	9.93	214.82	204.89	52.06	27.38	749,658	1.24	1.89
	Testing subset							
Uw	0.81	1.15	0.34	0.96	0.14	0.02	0.35	− 1.55
Ur	2.20	2.40	0.20	2.31	0.10	0.01	− 0.11	− 1.99
Us	3.15	3.55	0.40	3.35	0.20	0.04	0.03	− 2.00
L	10.01	30.00	19.99	20.86	5.48	29.98	− 0.19	− 1.05
W	10.00	30.00	20.00	21.00	5.49	30.16	− 0.20	− 1.06
H	4.00	15.00	11.00	8.89	3.14	9.88	0.27	− 1.08
O	0.00	360	360	179	104	10,901	0.01	− 1.20
Ws	0.00	0.80	0.80	0.40	0.23	0.05	− 0.06	− 1.21
We	0.00	0.80	0.80	0.40	0.23	0.05	− 0.02	− 1.14
Wn	0.00	0.80	0.80	0.40	0.23	0.05	0.00	− 1.17
Ww	0.00	0.80	0.80	0.41	0.23	0.05	− 0.07	− 1.17
Ug	0.01	1.20	1.19	0.61	0.34	0.11	− 0.08	− 1.16
SHGC	0.01	0.99	0.98	0.42	0.28	0.08	0.35	− 1.11
VT	0.01	0.99	0.98	0.51	0.29	0.09	− 0.04	− 1.25
Heat	8.00	12.00	4.00	9.97	1.42	2.03	0.03	− 1.32
Cool	18.00	28.00	10.00	22.62	3.07	9.40	0.10	− 1.17
EC	12.02	156.28	144.26	53.01	25.74	662,535	1.05	0.84

**Table 2:**
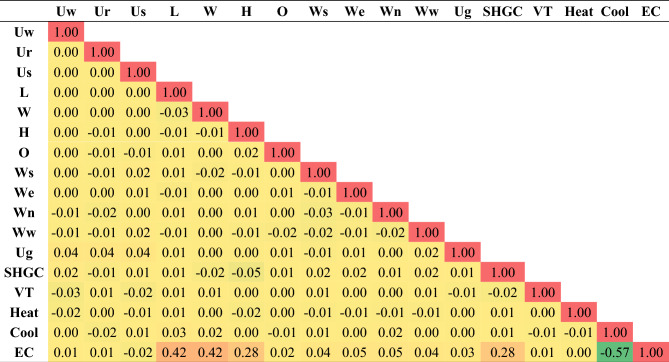
Pearson correlation matrix.

### Model (1): (GP) technique

The developed GP model has 5 levels of complexity. The population size was 10,000, survivor size was 3000, and number of generations was 150. Row database is used directly without any pre-processing to generate the predictive model. The generated model is a closed form formula that presented in Eq. 5. The performance indicators for this model are (RMSE = 3970), (ERROR% = 7.6%) and (R^2^ = 0.95).

5 $${\text{EC}} = \frac{{50000 {\text{W}}}}{{{\text{Cool}}^{2} }} + \left( {\frac{{50000 {\text{W}}}}{{{\text{Cool}}^{2} }} + {\text{H}}^{2} ({\text{SHGC}}^{2} + 1)} \right)\left( {{\text{L}} + \left( {{\text{H}} + 1} \right){\text{SHGC}} - \frac{{\text{W}}}{{\text{H}}}} \right)$$

### Model (2) (EPR) technique

The developed EPR model was limited to the quadratic level. For 16 parameters, there are 137 possible terms (120 + 16 + 1 = 137) as follows:

6 $$\sum\limits_{i = 1}^{i = 16} {\mathop \sum \limits_{j = 1}^{j = 16} } X_{i} \cdot X_{j} + \mathop \sum \limits_{i = 1}^{i = 16} X_{i} + C$$

Row database is used directly without any pre-processing to generate the predictive model. The most influencing 19 terms were determined using the GA technique, and the output is a polynomial formula that is shown in Eq. (7).

The performance indicators for this model are (RMSE = 1101), (ERROR% = 2.1%) and (R^2^ = 0.97).

7 $${\text{Fc}} = 4235 {\text{L}} + 4354{\text{ W}} + 5055 {\text{H}} + 19565 {\text{SHGC}} - 2998 {\text{Cool}} + 77 {\text{L}} \times {\text{W}} + 72 {\text{L}} \times {\text{H}} + 74 {\text{W}} \times {\text{H}} + 606 {\text{L}} \times {\text{SHGC}} + 632 {\text{W}} \times {\text{SHGC}} + 3020 {\text{H}} \times {\text{SHGC}} - 201 {\text{L}} \times {\text{Cool}} - 206{\text{ W}} \times {\text{Cool}} - 317 {\text{H}} \times {\text{Cool}} - 2050 {\text{SHGC}} \times {\text{Cool}} + 17.2{\text{H}}^{2} + 1950 {\text{SHGC}}^{2} + 219{\text{ Cool}}^{2} - 34690$$

### Model (3): (ANN) technique

**Figure 3:**
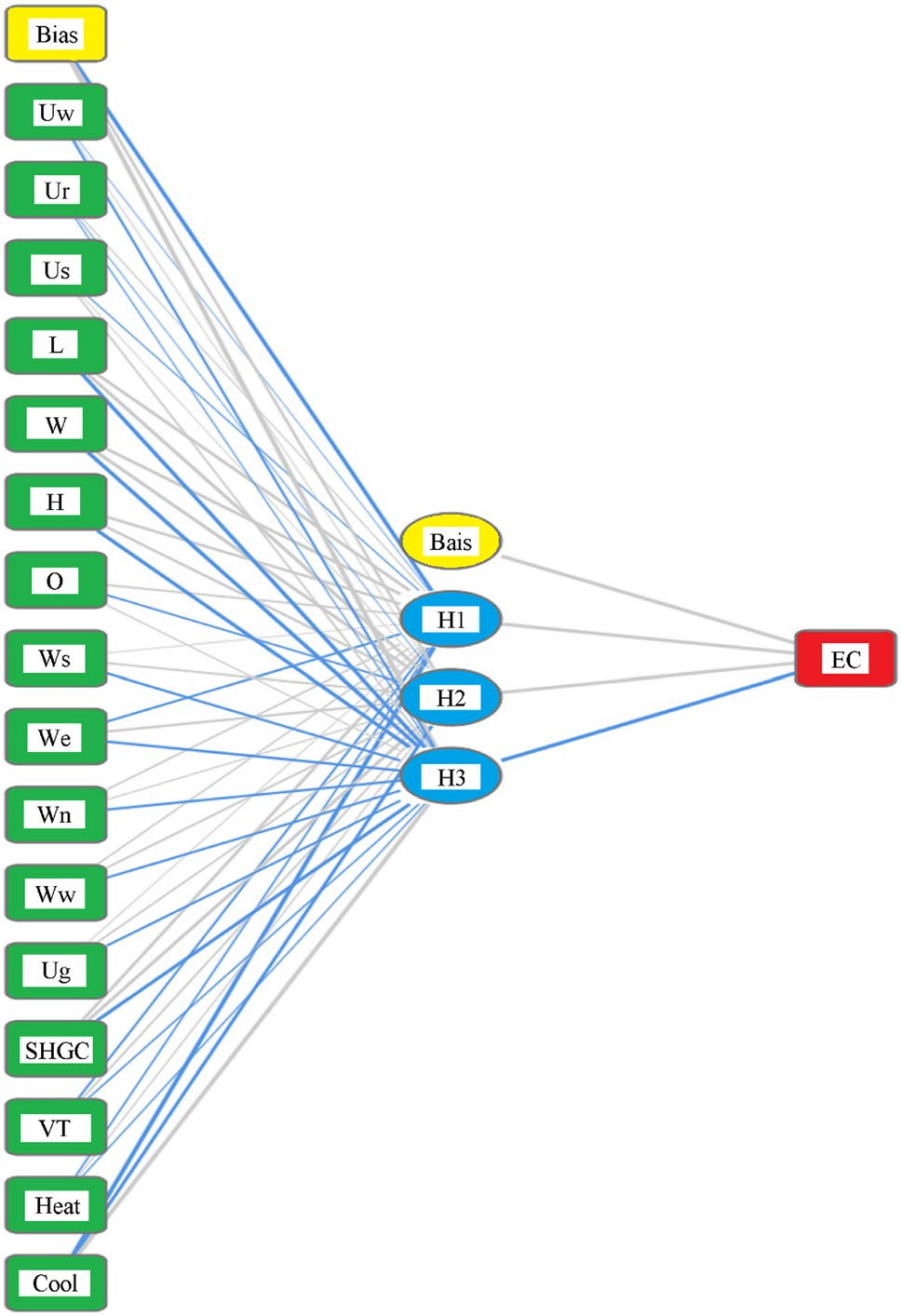
Architecture of the developed ANN model.

**Figure 4:**
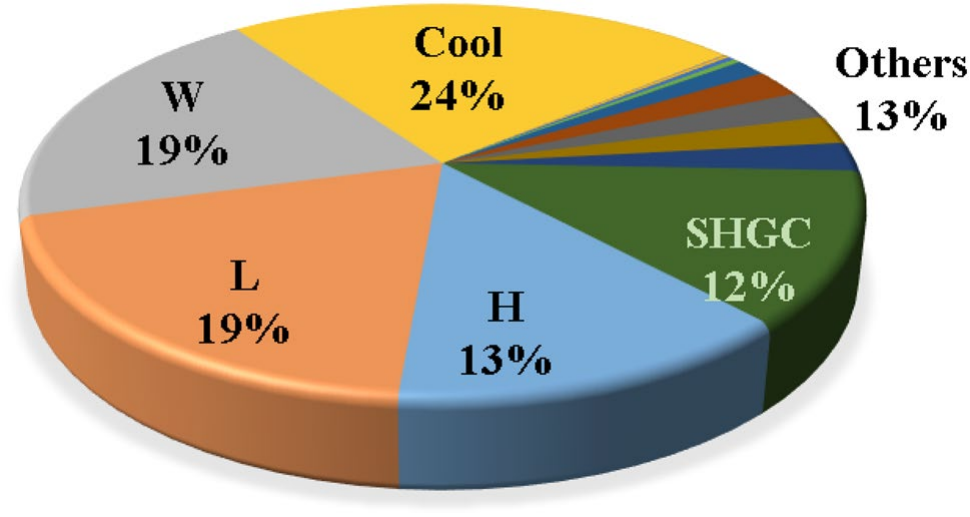
Relative importance of parameters.

The developed model architecture was restricted to 16 neurons in the input layer (number of considered parameters) and one neuron in the output layer (only one output), while the number of neurons in the hidden layer started with only one and gradually increased to three neurons to enhance the performance of the model. Unlike the GP, in the EPR models, the database was scaled using the standardization method (dividing the parameter value by the parameter standard deviation). This scaling is reversed after prediction to put the predicted value back in the normal scale. Hyperbolic Tangent (Hyper Tan) was selected as activation function and “Back Propagation (BP)” training technique were considered. The training continues until the error difference between iterations is less than 0.001. The architecture of the final model is illustrated in Fig. 3. Figure 4 presents the relative importance of each parameter and was calculated based on the summation of absolute weights of links, which are connected to the input neuron of each considered parameter. The trained matrix of weights is listed in Table 3.

**Table 3:** Matrix of weights for the developed ANN model.

	Hidden Layer			
	H(1:1)	H(1:2)	H(1:3)	EC
Input layer				
(Bias)	0.60	− 0.28	0.21	
Uw	0.00	− 0.08	0.00	
Ur	0.00	0.29	0.00	
Us	0.01	0.06	0.00	
L	0.00	0.09	− 0.14	
W	0.00	0.03	− 0.14	
H	− 0.25	0.23	− 0.01	
O	0.00	− 0.46	0.00	
Ws	− 0.07	− 0.51	0.01	
We	− 0.08	− 0.23	0.01	
Wn	− 0.08	0.06	0.01	
Ww	− 0.07	0.17	0.01	
Ug	0.01	0.28	− 0.01	
SHGC	− 0.26	− 0.16	0.00	
VT	0.00	− 0.24	0.00	
Heat	0.00	− 0.01	0.00	
Cool	0.11	0.63	0.15	
Hidden layer				
(Bias)				− 0.18
H(1:1)				− 0.63
H(1:2)				0.00
H(1:3)				− 1.43

The performance indicators for this model are (RMSE = 4375), (ERROR% = 8.4%) and (R^2^ = 0.97).

## Results

**Figure 5:**
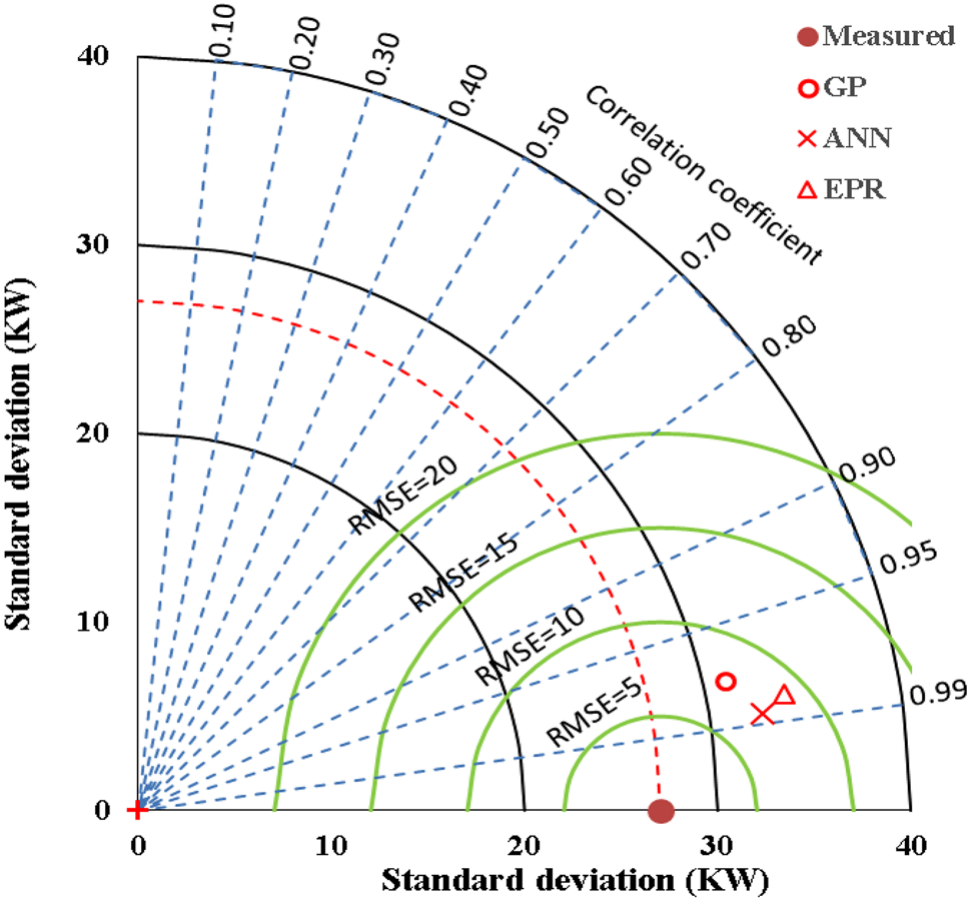
Comparing the developed models’ performance using Taylor’s chart.

**Figure 6:**
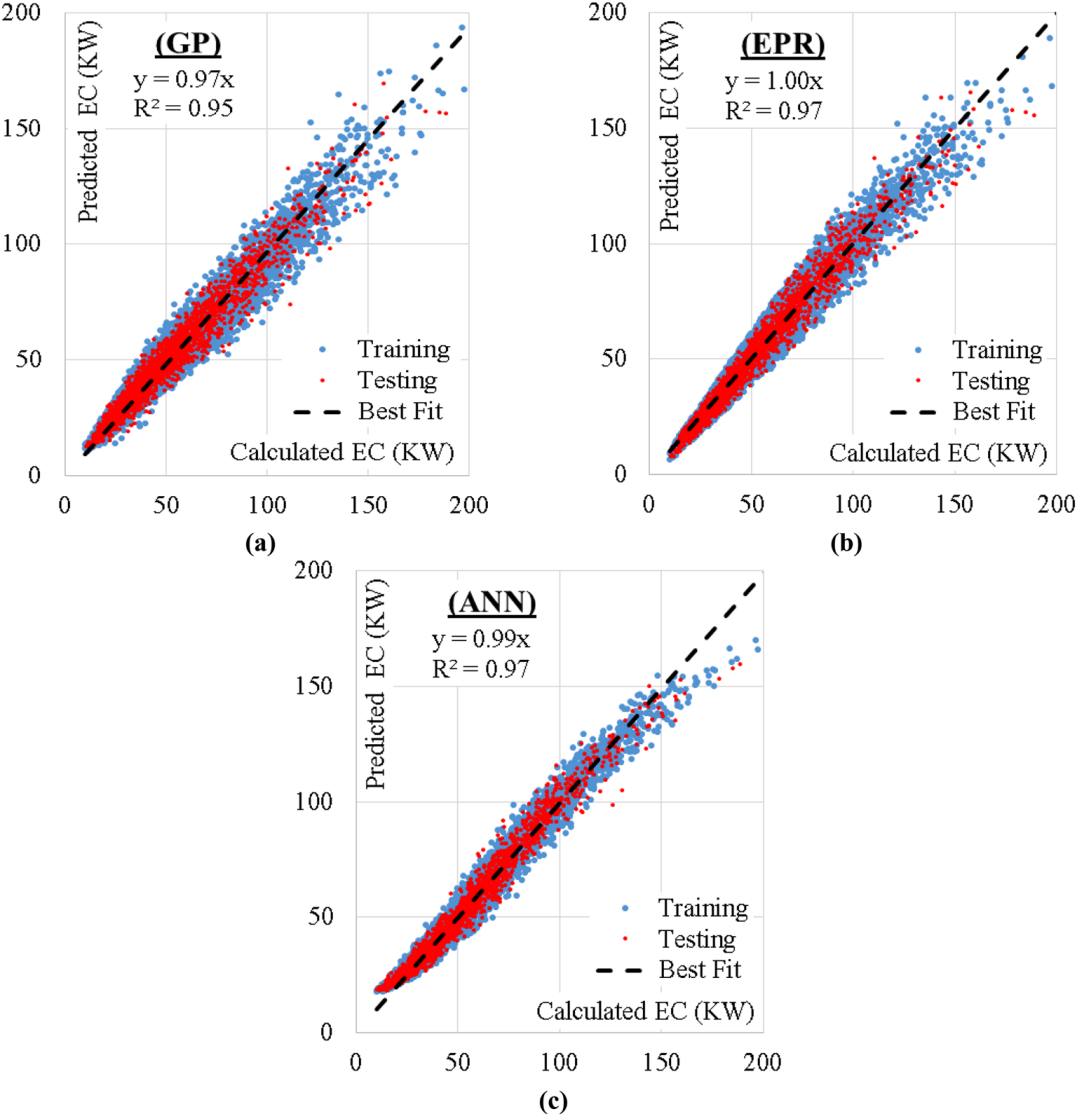
Relation between predicted and calculated (EC) values using the developed models.

The performance of the developed predictive models is summarized in Table 4. Figure 5 compares the developed models’ performance graphically using Taylor’s chart. Finally, Fig. 6 illustrates the relationship between the calculated (EC) values and the corresponding predicted values using the three developed models.

**Table 4:** Performance indicators of the developed models.

Tech	Model	Training set			Validation set		
		RMSE	Error%	R^2^	RMSE	Error%	R^2^
GP	Equation (5)	3970	7.6%	0.95	4296	8.2%	0.93
EPR	Equation (7)	1101	2.1%	0.97	1124	2.3%	0.96
ANN	Figure 3, Table 3	4375	8.4%	0.97	4557	8.8%	0.95

## Discussion

The implications of employing AI techniques in estimating energy consumption lie in its potential for optimizing and managing energy resources. AI models can exploit extensive amounts of data from several sources, including building characteristics, weather patterns, historic energy conservation, occupants’ performance, and energy simulation models. By learning patterns and relationships within this data, AI models can present more precise and accurate energy consumption estimation associated to conventional estimating approaches. AI models enable real-time supervising and assessment of energy consumption models, acknowledging for appropriate interventions and alterations to enhance energy usage. This potential is especially crucial for controlling energy demand. The statistical analysis of the utilized database that was summarized in Table 1 indicated several points as follows:

- The statistical characteristics of both training and testing subsets are very close, which assures the validity of the testing subsets.
- The ratio (S.D./Mean) is a good measurement for the scattering of the collected values of each parameter. Accordingly, Table 1 indicates uniform scattering range of all parameters except for Uw, Ur, and Us. Figure 1 also assured the gapped distribution of these parameters.
- The correlation coefficients listed in Table 2 illustrate the strong direct correlation between EC and the building dimensions (L, W and H) and Solar Heat Gain Coefficient (SHGC), as well as its strong inverse correlation with the target inside temperature in summer (Cool). These correlations make perfect sense; the bigger the building and the higher the heat gain, the higher the energy consumption needed to cool it. Also, the less the target inside temperature, the higher the EC required to achieve it, especially in summer.
- Because the utilized database was generated for semiarid and arid desert climate (warm in winter and hot in summer), the target inside temperature in summer (cool) has a much stronger influence on the energy consumption (EC) than the target inside temperature in winter (heat). The developed predictive models present different alternatives with different performances, advantages, and disadvantages. From an accuracy point of view, the ANN and GP models shared almost the same level of accuracy (about 92%) while the EPR model had a slightly better accuracy of 98%. From an applicability point of view, GP and EPR offered a closed form formula that could be used manually or embedded in a software, while the ANN model presented a matrix of weights with pre-standardization and post de-standardization, which is very complicated to be manually applied and needs a special software to implement it. Only five parameters of the considered 16 were used in the GP and EPR models. These parameters are L, W, H, SHGC and cool. Additionally, the relative importance chart from the ANN model in Fig. 3 showed that these five parameters present 87% of the total impact on the EC. These notes match the previous ones from the statistical analysis.

The effectiveness of AI models heavily relies on the quality and availability of data. Limited or low-quality data on building characteristics, occupant behavior, equipment usage, home appliances, energy consumption patterns, or environmental factors may hinder the accuracy and generalizability of the energy estimation model. Selecting the most appropriate technique among GP, ANN, and EPR depends on the specific characteristics of the data and the desired level of interpretability. The applicability of the energy estimation model developed using GP, ANN, and EPR may vary across different geographical regions, building type, and climate conditions. Factors such as building regulations and cultural differences may limit the transferability of the model to new contexts, requiring validation and adaptation for each specific use case. Addressing these limitations requires careful consideration of data quality, model complexity, generalization performance, computational resources, algorithmic biases, and transferability throughout the research process.

## Conclusions and future recommendations

This study presented three AI predictive models to estimate the building energy consumption during the conceptual design stage. The GP, EPR, and ANN techniques were applied on a generated database from previous research. The utilized database was based on data from the semiarid and arid desert climate and contained 16 parameters including building dimensions (L, W & H), orientation (O), thermal conductivity of walls, roofs, slabs on grade and windows (Uw, Ur, Us & Ug), window ratio for each façade (Wn, Ws, We & Ww), window glass properties (SHGC and VT), and finally the target inside temperature in summer and winter (cool and heat). The results of this study could be summarized in the following points:

- The developed EPR model presented the most accurate and applicable alternative with an accuracy of 98%, while the GP model presented a slightly less accurate alternative of 92.5%, but it is still considered a user-friendly closed-form equation. Finally, the ANN model presented a “Black Box” alternative with the same accuracy of the GP model.
- All the developed predictive models and the statistical analysis showed that only five parameters dominate the building energy consumption (EC), which are the building dimensions (L, W and H) besides the Solar Heating Glass Coefficient (SHGC) and the target inside temperature in summer (Cool). These five parameters have 87% of the total impact on the (EC), while the remaining nine parameters shared 13%.
- The target inside temperature in summer (Cool) was the most influential parameter on the (EC), with a relative importance of 25% because the utilized database was generated for semiarid and arid desert climate (warm in winter and hot in summer).
- Similar to all AI predictive models, the developed models are valid only within the considered range of parameter values. Beyond this range, the models must be verified.

This study opens paths for investigating AI methods for estimating building energy in semiarid and arid desert climate. Suggestions for future studies include incorporating additional data sources integrating weather data and occupant behavior profiles to improve energy simulation models. Merging AI methods like ANNs with physics-based building energy simulation models could influence the powers of both techniques for stronger results. Employing pre-trained AI models on comparable datasets from other desert areascould enhance the effectiveness and accuracy of the models. Incorporating such models utilized in this study with building management systems could be done for real-time energy consumption supervising, optimization, and identified suggestions for occupants. Finally, surveying procedures to improve the interpretability of the AI models for better understanding of the factors prompting energy consumption and facilitating targeted interferences for energy reserves.

## Data Availability

All data generated or analysed during this study are included in this published article.

## Abbreviations

AI: Artificial intelligence
GP: Genetic programming
EPR: Evolutionary polynomial regression
ANN: Artificial neural network
GA: Genetic algorithm
MAPE: Mean absolute percentage error
RMSE: Root mean squared error
MAE: Mean absolute error
R2: Coefficient of determination
L: Building length (m)
W: Building width (m)
H: Building height (m)
O: Building orientation with respect to north in (degrees)
Wn: Windows to wall ratio for North façade (%)
Ws: Windows to wall ratio for South façade (%)
We: Windows to wall ratio for East façade (%)
Ww: Windows to wall ratio for West façade (%)
Uw: Thermal conductance (U-value) of walls in (W/m^2^K)
Ur: Thermal conductance (U-value) of roof in (W/m^2^K)
Us: Thermal conductance (U-value) of slab on grade in (W/m^2^K)
Ug: Thermal conductance (U-value) of glass windows in (W/m^2^K)
SHGC: Solar Heat Gain Coefficient
VT: Visible Transmittance percent
Heat: The target temperature inside the building in winter in (°C)
Cool: The target temperature inside the building in summer (Cool) in (°C)
EC: The calculated electricity consumption value in (Watts)
